# Efficacy and prognostic analysis of chemoradiotherapy in patients with thoracic esophageal squamous carcinoma with cervical lymph nodal metastasis alone

**DOI:** 10.1186/s13014-014-0256-9

**Published:** 2014-11-26

**Authors:** Peng Zhang, Mian Xi, Lei Zhao, Qiao-Qiao Li, Li-Ru He, Shi-Liang Liu, Jing-Xian Shen, Meng-Zhong Liu

**Affiliations:** State Key Laboratory of Oncology in South China, Collaborative Innovation Center for Cancer Medicine, Cancer Center, Sun Yat-sen University, Guangzhou, 510060 People’s Republic of China; Department of Radiation Oncology, Cancer Center, Sun Yat-sen University, Guangzhou, 510060 People’s Republic of China; Imaging Diagnosis and Interventional Center, Cancer Center, Sun Yat-sen University, Guangzhou, 510060 People’s Republic of China

**Keywords:** Chemoradiotherapy, Esophageal cancer, Prognosis, Cervical lymph nodal metastasis

## Abstract

**Background:**

The prognostic factors of thoracic esophageal squamous carcinoma with cervical lymph nodal metastasis (CLNM) have not been specifically investigated. This study was performed to analyze the efficacy and prognostic factors of chemoradiotherapy for thoracic esophageal carcinoma with CLNM alone.

**Methods:**

From 2002 to 2011, 139 patients with inoperable esophageal cancer who underwent chemoradiotherapy at the Sun Yat-Sen University were retrospectively analyzed. Median radiation doses were 60 Gy (range: 50–68 Gy). Univariate and multivariate analyses were performed to compare overall survival (OS) and progression-free survival (PFS).

**Results:**

The 1- and 3-year OS rates were 68.2% and 27.9%, respectively. The 1- and 3-year PFS rates were 51.9% and 20.1%, respectively. The multivariate analysis demonstrated that response to treatment, T stage, pathological grade, and laterality of cervical lymph nodal metastases were independent prognostic factors for thoracic esophageal carcinoma with CLNM.

**Conclusions:**

Concurrent chemoradiotherapy is an important and hopeful treatment option for patients with esophageal cancer with CLNM alone. Our study has revealed that response to treatment, T stage, pathological grade and laterality of cervical lymph nodal metastases are significant prognostic factors for long-term survival.

## Background

Esophageal cancer is an aggressive disease and has a liability of lymphatic and hematogenous dissemination. In addition, the prognosis of esophageal cancer is poor, and the 5-year survival rate of patients with distant metastasis is only 3% [1]. If distant metastasis occurs, the opportunity to operate is lost. For patients with inoperable esophageal cancer, chemoradiotherapy is the mainstay of treatment. Although cervical lymph nodal metastases were designated as stage IV according the 6th American Joint Committee on Cancer (AJCC) staging system for esophageal carcinoma, the long-term survival data of patients with stage IV disease is varied. It has been reported that long-term survival might be achievable in patients with cervical lymph nodal metastases [2].

Well-known prognostic factors of esophageal cancer are TNM stage, tumor length (for early stage esophageal cancer) [3], concurrent chemotherapy, histopathological grading, sex, and age [4,5]. The efficacy and prognosis of thoracic esophageal squamous carcinoma with cervical lymph nodal metastasis (CLNM) alone, have not been specifically determined.

Our study was performed to evaluate the efficacy and to explore the prognostic factors that are associated with overall survival (OS) and progression-free survival (PFS) in patients with thoracic esophageal squamous carcinoma with CLNM, who were treated with chemoradiotherapy.

## Methods

### Patient population

Between February 2002 and December 2011, 139 patients who were diagnosed with esophageal cancer with cervical lymph nodal metastases alone at Sun Yat-sen University Cancer Center, were retrospectively studied. Each patient had histologically confirmed squamous cell carcinoma of the esophagus, was unable to undergo radical resection, and was treated with radiotherapy plus chemotherapy. The pretreatment evaluation consisted of endoscopy, barium esophagography and a computed tomography (CT) scan of the abdomen and thorax. Endoscopic ultrasound has only been available since 2006. Bone scans were performed if clinically indicated. The 6th AJCC staging system was used in this study. The criteria for lymph node positivity on the CT scan were either (1) short axis size >10 mm, (2) lymph node with infiltrative margin, or (3) central necrosis. According to the 6th AJCC classification, 54 patients with upper third thoracic cancer and cervical nodal metastases were staged as IVa, and the remaining 85 patients were staged as IVb.

This study was approved by the institutional review board (IRB) of the Cancer Center, Sun Yat-sen University. Written informed consent was obtained from all the patients in accordance with the regulations of the IRB.

### Treatment details

Radiotherapy was delivered with 6–10 MV photons once daily, five times a week (except weekends and public holidays), with a daily dose of 1.8-2 Gy. The total radiotherapy dose was <60 Gy in 45 patients and ≥60 Gy in 94 patients. The majority of patients (131) received three-dimensional conformal radiotherapy and the remaining eight patients received intensity-modulated radiotherapy (IMRT). The primary gross tumor volume (GTV-P) included esophageal lesions found on the radiograph and the gross tumor volume for the affected lymph nodes (GTV-N) was determined. The conformal primary tumor volume (CTV-P) included the GTV-P with a 3-cm margin (craniocaudal direction) and a 0.5-cm margin (lateral and anterior–posterior directions). The CTV of the upper-third of the squamous cell carcinoma (SCC) encompassed the bilateral supraclavicular region.

Among the 139 patients, 29 received induction chemotherapy plus concurrent chemoradiotherapy. The other 110 patients received concurrent chemoradiotherapy. The induction chemotherapy was two cycles of docetaxel (70 mg/m^2^) and cisplatin (80 mg/m^2^). Fifty patients were treated with two cycles of docetaxel (60 mg/m^2^) and cisplatin (80 mg/m^2^) delivered on days 1 and 22 of radiotherapy [6]. Twenty-two patients received at least four cycles of docetaxel (30 mg/m^2^) and cisplatin (35 mg/m^2^) per week. Another 44 were treated with two cycles of 60 mg/m^2^ of cisplatin administered on days 1 and 29 and 300 mg/m^2^/24 h of 5-FU administered on days 1–3 and days 29–31 [7]. The remaining 23 patients received other regimens of chemotherapy such as navelbine plus cisplatin.

### Toxicity and response assessment

The evaluation of acute treatment toxicities consisted of history and physical examination, documentation of performance status, complete blood count, and toxicities scoring. A routine barium swallow was performed by the radiation oncologist at doses of 20, 40 and 60 Gy. One day before starting the next chemotherapy cycle, a full blood count and serum chemistry was carried out. The acute toxicity of radiotherapy and chemotherapy were evaluated according to CTC version 2.0. The treatment effect was assessed at one month after finishing treatment, and included physical examination and history, endoscopy, and barium swallow. Treatment response was according to the guidelines for solid tumors [8] as follows: a complete response (CR) was defined as the complete disappearance of clinically detectable tumor masses; a partial response (PR) required a >30% decrease in the sum longest diameter of tumor for at least 1 month; the appearance of new lesions or a 20% increase in the sum longest diameter of an existing tumor was reported as progressive disease (PD). Neither PR nor PD criteria met the standards for stable disease (SD).

### Follow-up and statistical analysis

Overall survival and progression-free survival were calculated for each potential prognostic factor with the Kaplan–Meier method and were measured from the first day of diagnosis or censored at the date of the last follow-up. The log-rank test was used for testing significance, and the level of statistical significance was set at *p* <0.05. Multivariate analyses were performed by Cox regression. The last follow-up evaluation was performed in April 2013. All statistical analyses were performed using SPSS 16.0 software (SPSS Inc., Chicago, IL, USA).

## Results

### Patient characteristics

Between 2002 and 2011, 139 consecutive patients with inoperable esophageal squamous cell carcinoma were studied. The demographic data of the patients are shown in Table 1. There were 115 (82.7%) male and 24 (17.3%) female patients with a median age of 63 years (range: 34–86). The median follow-up was 23 months (range: 2–117 months).

**Table 1:** **Patient characteristics**

**Characteristics**	**No.**	**%**
Sex		
Male	115	82.7
Female	24	17.3
Histological grading		
G1	12	8.6
G2	61	43.9
G3/4	38	27.3
Gx	28	20.1
Tumor site		
Proximal	54	38.8
Medial	75	54.0
Distal	10	7.2
Tumor legngth		
≤ 5 cm	65	46.8
> 5 cm	74	53.2
T stage		
T1/2	17	12.2
T3	68	48.9
T4	54	38.8
M stage		
M1a	54	38.8
M1b	85	61.2
Laterality		
Unilateral CLNM	106	76.2
Bilateral CLNM	33	23.7
RT dose		
< 60 Gy	45	32.4
≥ 60Gy	94	67.6
Chemotherapy mode		
Induced CT+RCT	29	20.9
RCT	110	79.1
Concurrent chemotherapy		
Cisplatin+5-Fu	44	31.7
Cisplatin+ docetaxel	72	51.8
Other	23	16.5
Response to treatment		
CR	48	34.5
Non-CR	91	65.5

RT: radiotherapy; CT: chemotherapy; RCT: radiochemotherapy; CLNM: cervical lymph nodal metastases.

### Response to treatment and survival

Of the 139 eligible patients, 48 (34.5%) achieved a CR; and 71 (51.1%) patients demonstrated a PR according to the guidelines for solid tumors, which resulted in a response rate of 76.3%. Nine (6.5%) patients achieved SD and the remaining 11 patients achieved PD (7.9%).

**Figure 1:**
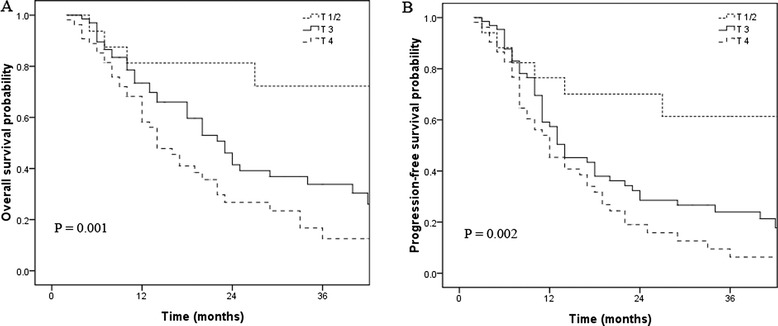
**Overall survival (A) and progression-free survival (B) according to clinical T stage.**

The 1- 2- and 3-year survival rates were 68.2%, 39.1%, and 27.9%, respectively; and the 1, 2, and 3 years of progression-free survival rates were 51.9%, 29.8%, and 20.1%, respectively (Figure 1).

### Treatment-related toxicity

Most treatment-related and documented acute toxicities were grades 1 and 2. The most common grades 3 and 4 toxicities were leukopenia (48 patients; 34.5%) and gastrointestinal toxicity (15 patients; 10.8%). Laryngeal edema occurred in one patient. No therapy-related deaths occurred. Acute treatment-related toxicities were evaluated based on the CTC 3.0 and are listed in Table 2.

**Table 2:** **Acute toxicity according to CTC v 3.0**

**Toxicity**	**Grade 0**	**Grade 1**	**Grade 2**	**Grade 3**	**Grade 4**
	**n (%)**	**n (%)**	**n (%)**	**n (%)**	**n (%)**
Anemia	27 (19.4)	53 (38.1)	52 (37.4)	7 (5.0)	0 (0)
Leukocytopenia	17 (12.2)	44 (31.7)	60 (43.1)	30 (21.6)	18 (12.9)
Thrombopenia	50 (40.0)	45 (32.4)	24 (17.3)	18 (12.9)	2 (1.4)
Gastrointestinal	17 (12.2)	75 (54.0)	32 (23.0)	14 (10.1)	1 (0.7)

Fifteen patients (10.8%) developed an esophagostenosis in subsequent follow-up. Radiotherapy-related lung disease was diagnosed in seven patients (5.0%). One patient developed paralysis of the vocal cord.

### Association of survival and clinicopathologic factors

Univariate analyses were performed for each prognostic factor. Response to treatment (*p* <0.001), pathological grade (*p* =0.047), T stage (*p* =0.001), laterality of cervical lymph nodal metastases (*p* =0.009), and chemotherapy regimen (*p* =0.003) had an effect on overall survival rate (Table 2). Gender, primary tumor length, radiotherapy dose, M stage and introduction chemotherapy had no statistically significant impact on overall survival; it is worth noting that tumor location approached statistical significance (*p* =0.055).

In the univariate analyses of the PFS, response to treatment (*p* <0.001), pathological grade (*p* =0.038), T stage (*p* =0.002), laterality of cervical lymph nodal metastases (*p* =0.006), and chemotherapy regimen (*p* =0.034) had an effect on progression-free survival rate. Sex, primary tumor length, radiotherapy dose, M stage, tumor location, and introduction chemotherapy had no statistically significant impact on progression-free survival.

**Figure 2:**
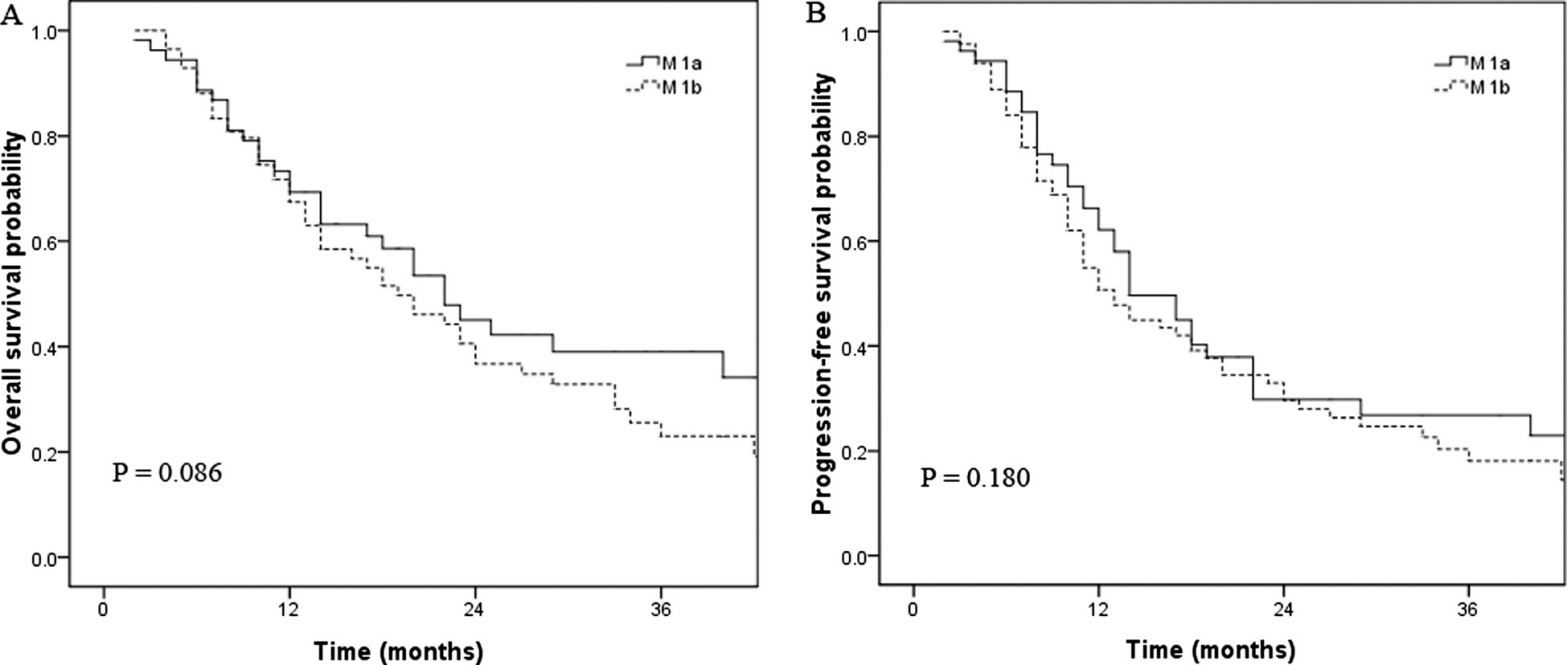
**Overall survival (A) and progression-free survival (B) according to M stage.**

The 3-year OS of the patients with grade IVa disease (primary lesions located in the upper-third esophagus) was 35.9%, which was better than that of the patients with grade IVb disease (primary lesions located in the middle- and lower-third of the esophagus), for which the 3-year OS was 23.0%. However, this difference was not statistically significant (*p* =0.086; Figure 2).

**Figure 3:**
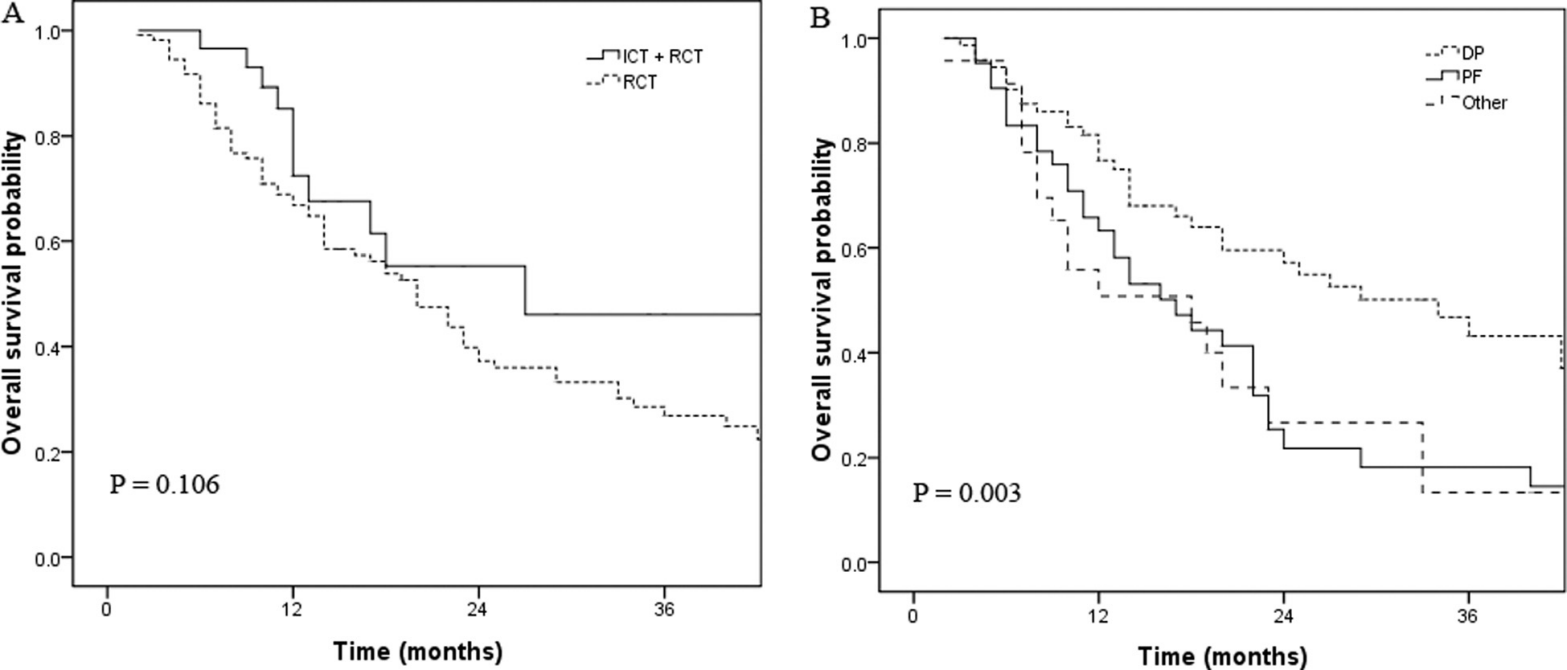
**Overall survival according to chemotherapy mode (A) and concurrent CRT regimen (B).**

Patients who received induced chemotherapy did not show a benefit in OS (p =0.106; Figure 3A) and PFS (p =0.253) compared with patients who received chemoradiotherapy.

After treatment, patients with a CR had an obviously better prognosis than non-CR patients. The 3-year OS of patients with a CR was 53.7%; whereas the 3-year OS of non-CR patients was only 20.1% (*p* <0.001). The 3-year PFS of CR and non-CR patients was 11.4% and 38.6%, respectively (*p* <0.001).

Patients with T1/2 disease had a better 3-year OS than stage T3 and T4 tumors (36.1% vs. 30.4% vs. 12.5% *p* =0.001). The 3-year PFS of patients with T1/T2 disease was significantly better than that of patients with T3 and T4 disease (30.7% vs. 21.3% vs. 6.3%; *p* =0.002).

Patients with unilateral cervical lymph nodal metastases had a better 3-year OS (33.5%) and a better PFS (26.4%) compared with those with bilateral metastases (3-year OS rate, 14.8%, *p* =0.009; 3-year PFS rate, 4.3%, *p* =0.006).

The 3-year OS and PFS of patients who received cisplatin and docetaxel were 37% and 22.7%, respectively. The 3-year OS and PFS of patients who received cisplatin and 5-FU were 14.5% and 13.1%, respectively (*p* =0.003), while the 3-year OS and PFS rates of patients who received other regimens were 13.3% and 11.0%, respectively (*p* =0.034).

### Multivariate analysis

Multivariate analysis was performed for OS, including factors found to be significant on univariate analysis. Additionally, a backward stepwise Cox regression analysis was performed. According to the analysis, response to treatment (*p* <0.001), clinical T stage (*p* =0.007), pathological grade (*p* =0.002), and laterality of cervical lymph nodal metastases (*p* =0.023) were independent prognostic factors for OS. Concurrent chemotherapy (*p* =0.117) was not statistically significant.

## Discussion

Cervical lymph nodal metastases (CLNM) are not rare in thoracic esophageal squamous carcinoma. Huang *et al*. reported the pattern of thoracic SCC lymph nodal metastases after esophagectomy. In their study, the rates of CLNM in patients with upper, middle and lower thoracic tumors were 16.7% (9/54), 4.0% (27/680) and 1.0% (5/343), respectively [9]. It is worth pointing out that their cases all received surgery and there is an inevitable operation selection bias. The CLNM rates may be higher for patients with inoperable SCC treated with chemoradiotherapy in consideration of more advanced staging.

Previous literature reporting the prognosis of thoracic esophageal squamous carcinoma with CLNM (stage IV) is conflicting [2,10]. Shimada *et al*. retrospectively analyzed 88 patients who were diagnosed with thoracic esophageal cancer with CLNM and reported that the 5-year OS rate of these patients was 26% [2]. The Japanese Society for Esophageal Diseases has divided cervical nodes into four groups: cervical paraesophageal nodes, deep cervical nodes, retropharyngeal nodes, and supraclavicular nodes, and involvement of the cervical paraesophageal nodes was defined as stage N1 in the case of cancers of the upper third of the esophagus [11]. According to the Chinese non-operative stage of esophageal cancer, patients with CLNM are considered to be stage N1 (cervical esophageal cancer) or N2 (thoracic esophageal cancer) [12]. Although patients with CLNM only were graded as stage IV according to the AJCC 6th TNM system, we still adopted the more radical treatment to expect a better prognosis.

We performed a comprehensive assessment of the efficacy and prognostic factors of chemoradiotherapy for thoracic esophageal squamous carcinoma with CLNM only, and have shown that the prognosis of these patients is not dismal, despite it being stage IV. In our study, the 3-year OS and PFS rates of these patients were 27.9% and 20.1%, respectively. The multivariate analysis showed that response to treatment (*p* <0.001), clinical T stage (*p* =0.007), pathological grade (*p* =0.002), and laterality of cervical lymph nodal metastases (*p* =0.023) have a significant bearing on overall survival. The main pattern of treatment failure is still the local recurrence (61/139, 43.9%).

Locoregional control remains the major problem in patients with SCC treated with chemoradiotherapy. In our study, patients who achieved a complete response (CR) had an obviously better survival than those who did not (non-CR), which is consistent with previous literature [13,14]. Ohtsu *et al*. reported that a CR rate of 33% and a 3-year survival rate of 23% were achieved in patients with unresectable T4 tumors and/or M1 LYM (lymphatic metastasis) disease. In this study, the chemoradiotherapy consisted of a total radiation dose of 60 Gy with concurrent fluorouracil (400 mg/m^2^) and cisplatin (40 mg/m^2^), followed by two courses of fluorouracil (800 mg/m^2^/24 hours for 5 days) and cisplatin (80 mg/m^2^ on day 1) [14].

The primary lesion T stage also has a bearing on the prognosis. In our study, the survival of patients with T1/2 disease is better than that of those with a T3 lesion, and the prognosis of patients with T3 disease is better than that of those with T4 lesions. Increasing depth of tumor invasion is associated with the presence of lymphatic dissemination and thus leads to the unfavorable prognosis [15]. The same results were achieved in the analysis of the PFS. In accordance with our results, Kaneko *et al*. reported that the prognosis of patients with T3 lesions was better than that of those with T4 tumors with squamous cell carcinoma of the esophagus and concluded that the efficacy and survival of patients treated with chemoradiotherapy are related to the T stage [16].

It is worth pointing out that the laterality of the CLNM has a bearing on the prognosis of patients with thoracic esophageal squamous carcinoma and CLNM (*p* =0.023). The results may be attributed to the fact that patients with unilateral CLNM have an earlier staging than patients with bilateral CLNM. Skip metastasis may be another factor that influences prognosis. Prenzel *et al*. reported that skip metastases are associated with better 5-year survival rates and have a higher rate in cancer of the middle and upper thirds of the esophagus [17].

The 7th edition AJCC staging system shows that the tumor location is a component of staging and that the prognosis of the lower third of esophageal cancer is better than that of the upper and middle thirds of the esophagus [18]. In this regard, our data did not reveal a significant impact for tumor location. However, there were trends observed (*p* =0.055). This result may be owing to the relatively small number of patients. On the other hand, the patients with stage IVa disease (primary lesions located in the upper third of the esophagus) had a better (but not significant) prognosis than patients with stage IVb disease (primary lesions located in the middle and lower thirds of the esophagus), which is in accordance with the 6th AJCC staging system.

The RTOG 85–01 clinical trial demonstrated that combined chemoradiotherapy has a significant advantage over radiotherapy only, in patients with esophageal cancer. But, the concurrent chemotherapy regimen is far from conclusive [19]. In a phase I trial, Day *et al*. reported that docetaxel, cisplatin and concurrent radical radiotherapy is safe and efficient for locally advanced esophageal cancer with a complete response of 33%. The progression-free survival at 2 years was 49.7% and at 5 years was 26.5% [20]. In addition, a phase III trial reported that the pathological complete response (pCR) of preoperative chemoradiotherapy for esophageal carcinoma was 29% and the pCR of esophageal squamous-cell carcinoma was 49%. These findings reveal that the concurrent carboplatin plus paclitaxel regimen is rather effective compared with the previous regimen [21]. In a multicenter phase II trial, a pCR rate of 38% was achieved in patients with esophageal squamous cell cancer, while the pCR of patients with adenocarcinoma was only 16% [22]. In the univariate analyses of our study, the chemotherapy regimen had a significant influence on the OS and PFS (*p* =0.003 and 0.034, respectively). But, the chemotherapy regimen did not reach significance in the multivariate analysis (*p* =0.117). We deduced that this result is chiefly because of the small number of patients included in our study.

We did not observe a survival benefit for higher radiation dose when compared with lower radiation dose, which is in accordance with the results of the INT0123 study. The INT0123 trial reported that a higher radiation dose did not increase survival or local/regional control in patients with M0 esophageal cancer (by increasing the radiation dose from 50.4 Gy to 64.8 Gy) with a concurrent chemotherapy regimen of cisplatin/5-FU [23]. In a phase I/II trial, Wu *et al*. reported the treatment effect of patients with esophageal squamous cell carcinoma who received three-dimensional conformal radiation therapy. The 2-year local disease progression-free rate and distant metastasis-free rate were 36% and 56%, respectively, which were disappointing [24]. Our results also showed that a higher radiation dose (>60 Gy) did not increase the overall survival or progression-free survival in the M1 patients (*p* =0.107 and 0.605, respectively).

Although numerous studies have indicated that tumor length influences the prognosis of esophageal cancer [3,25], the patients in these studies had an earlier stage of disease. Yendamuri *et al*. reported that esophageal tumor length is independently associated with long-term survival, but found no statistical significance in the stage III patients [3]. The authors concluded that tumor length may be a better predictor of locoregional rather than distant control of disease. In our study, we only included the patients with stage IV disease. Our findings also reveal that primary tumor length does not have a significant impact in the prognostic analysis (*p* =0.911), which we attribute to more advanced stages and bulky lesions in our cohort.

The current study is limited by its retrospective design. Although the cases of cervical lymph nodal metastases in our study were based on radiology, the specificity of CT in detecting nodal metastases is 96.7%, and the accuracy is 76.6% for squamous cell carcinoma of the esophagus; here, nodal enlargement greater than 10 mm was considered indicative of involvement [26]. Considering these aspects of our study, longer follow-up and larger studies are needed in the future.

## Conclusion

The prognosis of esophageal cancer with CLNM only is not completely bleak. Concurrent chemoradiotherapy is an important and hopeful treatment option for these patients. The response to treatment, T stage, pathological grade and laterality of cervical lymph nodal metastases are significant prognostic factors for long-term survival.
